# Supplementary material for: Improving Oral Presentation Skills for Radiology Residents through Clinical Session Meetings in the Virtual World Second Life
Source: Int J Environ Res Public Health. 2023 Mar 8;20(6):4738. doi: 10.3390/ijerph20064738 (PMC10049172; doi:10.3390/ijerph20064738)
Supplement: Supplementary file 1 [file ijerph-20-04738-s001.zip › S1 QUESTIONNAIRE.pdf]

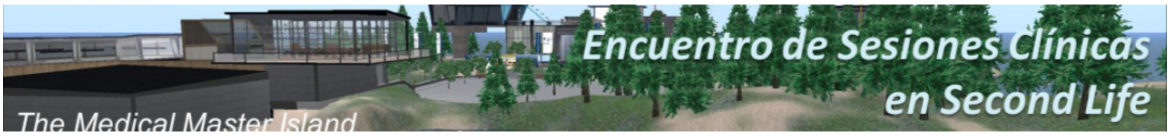

Clinical Session Meetings for radiology residents in Second Life

QUESTIONNAIRE

Please complete the following questions within the box, scoring from 1 to 5
(1 totally disagree, 5: totally agree)

1. The initiative seemed interesting to you ☐
2. The environment of the island seemed attractive to you ☐
3. The postgraduate building seemed suitable for you to carry out the course ☐
4. You knew Second Life before this experience ☐
5. You move through Second Life easily, without problems ☐
6. The tasks of creating and managing your avatar were easy ☐
7. Your computer met the requirements to run Second Life without problems ☐
8. Your Internet connection met the requirements to run Second Life without problems ☐
9. The contents of the meeting seemed adequate for your training as a resident ☐
10. The contents of the meeting were very difficult for your level of knowledge ☐
11. You would be willing to participate in another Second Life experience this year ☐
12. You would be willing to participate in another Second Life experience next years ☐
13. The intervention of the teachers was adequate ☐
14. The meeting program seemed interesting to you ☐
15. Your participation in the course was very active ☐
16. Contact with your colleagues in Second Life is very beneficial for your training ☐

Day 1. Introduction to Second Life, visit to the island and presentation of the meeting

The contents were interesting ☐
The extension of the contents was adequate ☐
I was able to follow the presentation(s) with ease ☐

Day 2. Conference: Clinical sessions in radiology

The contents were interesting ☐
The extension of the contents was adequate ☐
I was able to follow the presentation(s) with ease ☐

Day 3. Conference: How to speak in public

The contents were interesting ☐
The extension of the contents was adequate ☐
I was able to follow the presentation(s) with ease ☐

Location:
The Medical Master Island – postgraduate building.

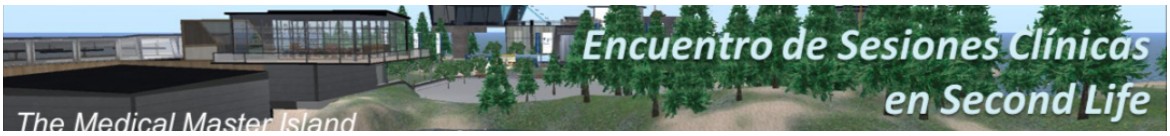

Clinical Session Meetings for radiology residents in Second Life

QUESTIONNAIRE

Please complete the following questions within the box, scoring from 1 to 5
(1 totally disagree, 5: totally agree)

Day 4. How to make proper PowerPoint presentations

The contents were interesting .... ☐
The extension of the contents was adequate........ ☐
I was able to follow the presentation(s) with ease........ ☐

Day 5. Presentation of clinical sessions: group 1

The contents were interesting .... ☐
The extension of the contents was adequate........ ☐
I was able to follow the presentation(s) with ease........ ☐

Day 6. Presentation of clinical sessions: group 2

The contents were interesting .... ☐
The extension of the contents was adequate........ ☐
I was able to follow the presentation(s) with ease........ ☐

Day 7. Presentation of clinical sessions: group 3

The contents were interesting .... ☐
The extension of the contents was adequate........ ☐
I was able to follow the presentation(s) with ease........ ☐

Day 8. Presentation of clinical sessions: group 4

The contents were interesting .... ☐
The extension of the contents was adequate........ ☐
I was able to follow the presentation(s) with ease........ ☐

Day 9. Presentation of clinical sessions: group 5

The contents were interesting .... ☐
The extension of the contents was adequate........ ☐
I was able to follow the presentation(s) with ease........ ☐

Day 10. Final conclusions – Closing

The contents were interesting .... ☐
The extension of the contents was adequate........ ☐
I was able to follow the presentation(s) with ease........ ☐

Location:

The Medical Master Island – postgraduate building.

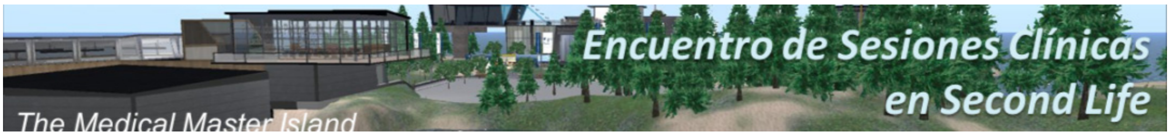

Clinical Session Meetings for radiology residents in Second Life

QUESTIONNAIRE

Important! Rating from 1 to 10 points

Finally, please rate the following aspects within the box, scoring from 1 to 10

The overall experience ☐

The organization of the project ☐

The island environment ☐

The educational contents ☐

The utility for your training ☐

The teachers ☐

The interaction with peers ☐

The synchronous sessions ☐

The tasks to do ☐

The connectivity to Second Life ☐

Open comments

If you want to add anything else, please use the box below

Location:

The Medical Master Island – postgraduate building.
