# Supplementary material for: Improving Oral Presentation Skills for Radiology Residents through Clinical Session Meetings in the Virtual World Second Life
Source: Int J Environ Res Public Health. 2023 Mar 8;20(6):4738. doi: 10.3390/ijerph20064738 (PMC10049172; doi:10.3390/ijerph20064738)
Supplement: Supplementary file 1 [file ijerph-20-04738-s001.zip › S2 OPEN COMMENTS.pdf]

Improving oral presentation skills for radiology residents through clinical session meetings into the virtual world Second Life
Open comments

Questionnaire 1. Delighted to have participated. I would do it again and have recommended it to my colleagues. It has been my first non-contact course and I see only advantages. The environment had me hooked, and especially the issue of doing it connected from home, but in real time. I would like to do more courses like this. And what I liked the most was the formative evaluation, a tool that I did not know as such but that intuitively seemed necessary and natural to me. I would like to bring this initiative to my department, but I know it is difficult!!! I think it would have been better if there had been more participation. But even so, I have taken advantage of it. Thank you!!!

Questionnaire 2. In general, I want to comment that it has been a very positive experience and that I hope we will organize another meeting again soon. I have discussed it with my fellow hospital residents, and I think if we organized another meeting they would sign up. Thank you so much for everything!!!!

Questionnaire 3. I found the possibilities offered by this environment very interesting, of which I was unaware of its educational usefulness. The audio is very immersive (hearing it depending on where the camera is, the sound that fades when the speaker moves away). Also very well achieved the feeling of being in a real classroom, but with the comfort of being at home. In any case, I miss the possibilities that being present offers you, especially when the speaker is good and takes advantage of these possibilities. In fact, I am recently giving some practical seminars at the university that I have prepared conscientiously and I feel much more comfortable standing up, looking at the faces of the students, moving among them, asking questions, standing next to them, adding video to the presentations… well. Summarizing. For a speaker who makes a flat presentation, without interactivity and without looking the audience in the eye, he will not notice the difference. But I have not felt comfortable talking alone in the room during my presentation, perhaps with the webcam activated and seeing our faces that feeling would have improved. However, it did seem to me that it facilitates the exchange of opinions between students, which I think is very beneficial for everyone. One of the most positive things I can get from the experience: sharing opinions with fellow radiologists from a distance. Let me know when you do a new course in Second Life!

Questionnaire 4. *No comment*

Questionnaire 5. I found it a very interesting experience. I would love to participate in future courses with content more related to radiology.

Questionnaire 6. *No comment*

Questionnaire 7. As we have all said in the course, it has helped us to improve our presentations (in fact, I already see the evolution in the PowerPoint presentations of my hospital colleagues), and it is totally recommended for our training, so, in new editions I hope that more residents, especially those of my service, be encouraged.

Questionnaire 8. *No comment*

Questionnaire 9. The initiative has seemed very good to me. The course, fabulous. At no time during our training in hospitals are we taught to give presentations, nor are we given constructive criticism on how to improve them, so I was delighted to be part of this course. The environment is very favorable and the atmosphere in the sessions has been very relaxed. I have loved being able to interact with residents from other hospitals. It has made me very happy to see his level, to realize how I am and what I have to improve. It is very gratifying to know that my hospital is not the worst in the world, even though it sometimes seems so.

Improving oral presentation skills for radiology residents through clinical session meetings into the virtual world Second Life

Open comments

Questionnaire 10. I had quite a few problems the first few days and the last day especially (the farewell day) with connectivity, but it was entirely my PC's fault, so I think the issue has nothing to do with the Second Life environment.

Questionnaire 11. Regarding the experience of the Second Life course, it seemed good to me, as at some point it was commented, it facilitates the acquisition of knowledge without the limitation of geographical mobility. For my particular taste, there have been many "face-to-face" days, it was commented that the possibility of shortening the sessions a bit and at the same time making them interactive, but it was said that it was not viable. I don't know if there is a possibility of making them blended, where we can review the sessions and topics with our own time availability and carry out 2-3 days of “face-to-face” review also in Second Life. Finally, I would like to thank the effort in which this course was carried out and reiterate that it has contributed knowledge to my training as a radiology resident.

Questionnaire 12. The periodicity must be two days a week because it becomes exhausting and threatens attendance. I would schedule thematic courses in second life for residents: thoracic, musculoskeletal and digestive radiology, emergency radiology. Endless possibilities to explore.

Questionnaire 13. I found it a complete and enriching experience in many ways, I have learned from everything! From what was the main objective to how to interact in environments as new as Second Life. The only part that has been difficult for me is -as has been evident- moving with the avatar across distances, the movements and actions have cost me a lot in some cases, otherwise I have nothing to say, everything has been very interesting and in a pleasant working environment that allowed learning. Undoubtedly, this environment has a potential that can be used in many ways. I hope to have the opportunity to participate again in activities of this type because they are -in addition to being fun- very instructive and a great source of learning.

Questionnaire 14. Thank you very much for the interest and effort you put into our training. It was a spectacular and highly motivating experience for me, so much so that I am looking forward to other Second Life courses.

Questionnaire 15. In general, it has been quite interesting. I would only put an inconvenience. The schedule, well Monday, Wednesday and Friday, 2 hours, is somewhat complicated to follow due to guards, study, courses, although in reality continuity and perseverance is what makes us learn. Unfortunately, in my case it has not been feasible, although I would have liked to attend all the sessions.

Questionnaire 16. It has been a very positive experience. From this course I have obtained several very useful concepts about oral presentations. To improve the course, I would shorten the number of daily meetings, perhaps increasing the hours per meeting, group more people presenting per day, and thus shorten the presentation time for each participant.

Questionnaire 17. I found it a very interesting meeting both for the topic discussed and for the platform used. For those of us who are just starting out, it is very important to learn how to do a good clinical session. In addition, the Second Life environment allows us to interact and learn from colleagues from different parts of the country quickly and easily. My main limitation was the Internet connection, since it was a problem for me when following the presentations (I couldn't see some slides because they didn't have time to load) and also because I heard the voice of the rest of the audience with some interruptions. participants. In general, I think it has been an enriching activity and I would participate again in future meetings.

Questionnaire 18. *No comment*.

Improving oral presentation skills for radiology residents through clinical session meetings into the virtual world Second Life
Open comments

Questionnaire 19. I have had the problem that I am not able to listen to the audio of the presentations that I could not attend, that is why I have not rated them. I found it a great experience and a very useful tool to exchange information, knowledge and at the same time meet people. All the best

Questionnaire 20. I really liked the experience in Second life, I will recommend it without a doubt, and I would love to be able to participate in other meetings like this one. Greetings and thank you.

Questionnaire 21. In this last rating, where I put 4 is simply because the platform is developing more and more, so the experience on it now is good but it will grow even more. And, furthermore, because new spaces are being created that will give rise to more meetings and more educational material. The only thing I miss is, in the undergraduate computers where x-rays appear, a mini-report where you transmit what the x-ray translates, because you don't know if what you see is true or not (maybe there is some way to see it but I couldn't get it , put only the clinic next to the x-ray). In summary, I am delighted with the activity given and received and I find the platform very interesting, the online route should be promoted more.

Questionnaire 22. I want to show my gratitude because I am frankly delighted with Second Life and I see it as a tool with great potential. I hope that meetings will continue to be organized and be part of them.

Questionnaire 23. I wanted to thank all the team that has been part of this experience, for letting me participate. When you start the residence you feel very lost (or at least I have felt that way) and having participated in Second Life in this meeting has improved my confidence in the presentations and in dealing with the rest of the residents, whom I don't know physically but it is as if I already knew them. I would really like you to continue organizing this type of event, perhaps a little more oriented towards radiology itself, because in addition to having fun studying, it is a good way to meet people and find out about the "news" that concern you as R1. Thank you very much for everything, I hope we meet again soon!
